# Development and validation of the Japanese version of the Hyperarousal Scale

**DOI:** 10.1186/s12888-022-04243-0

**Published:** 2022-09-19

**Authors:** Naoko Ayabe, Shun Nakajima, Isa Okajima, Ken Inada, Wataru Yamadera, Hidehisa Yamashita, Hisateru Tachimori, Yuichi Kamei, Masahiro Takeshima, Yuichi Inoue, Kazuo Mishima

**Affiliations:** 1 Department of Regional Studies and Humanities, Faculty of Education and Human Studies, Akita University, 1-1 Tegata-Gakuenmachi, Akita, 010-8502 Japan; 2 Department of Sleep-Wake Disorders, National Institute of Mental Health, National Center of Neurology and Psychiatry, 4-1-1 Ogawa-Higashi, Kodaira, Tokyo 187-8553 Japan; 3 National Center for Cognitive Behavior Therapy and Research, National Center of Neurology and Psychiatry, 4-1-1 Ogawa-Higashi, Kodaira, Tokyo 187-8553 Japan; 4 Department of Psychological Counseling, Faculty of Humanities, Tokyo Kasei University, 1-18-1 Kaga, Itabashi-ku, Tokyo, 173-8602 Japan; 5 Japan Somnology Center, Institute of Neuropsychiatry, 1-24-10 Yoyogi, Shibuya-ku, Tokyo, 151-0053 Japan; 6 Department of Psychiatry, Tokyo Women’s Medical University, 8-1 Kawadacho, Shinjuku-ku, Tokyo, 162-8666 Japan; 7 Department of Psychiatry, Kitasato University, School of Medicine, 1-15-1 Kitasato, Minami-ku, Sagamihara, Kanagawa, 252-0375 Japan; 8 Department of Psychiatry, The Jikei University Katsushika Medical Center, 6-41-2 Aoto, Katsushika-ku, Tokyo, 125-8506 Japan; 9 Minna no Sleep and Stress care Clinic, 2-1-2 Ushitahonmachi, Higashi-ku, Hiroshima-shi, Hiroshima, 732-0066 Japan; 10 Endowed Course for Health System Innovation, Keio University School of Medicine, 35 Shinanomachi, Shinjuku-ku, Tokyo, 160-8582 Japan; 11 Department of Clinical Data Science, Clinical Research & Education Promotion Division, National Center of Neurology and Psychiatry, 4-1-1 Ogawa-Higashi, Kodaira, Tokyo 187-8551 Japan; 12 Kamisuwa Hospital, 1-17-1 Oote, Suwa, Nagano, 392-0026 Japan; 13 Department of Neuropsychiatry, Akita University Graduate School of Medicine, 1-1-1 Hondo, Akita, Akita 010-8543 Japan; 14 International Institute for Integrative Sleep Medicine, University of Tsukuba, 1-1-1 Tennodai, Tsukuba, Ibaraki, 305-8575 Japan

**Keywords:** Hyperarousal, Insomnia, Depression, Hyperarousal scale

## Abstract

**Background:**

The objectives of this study were to develop a Japanese version of the Hyperarousal Scale (HAS-J) and investigate its factor structure, reliability, and validity, as well as to calculate a cutoff score for the HAS-J and assess different levels of hyperarousal in insomnia patients and community dwellers.

**Methods:**

We recruited 224 outpatients receiving insomnia treatment (56.3% women; mean age 51.7 ± 15.6 years) and 303 community dwellers aged 20 years or older (57.8% women; mean age 43.9 ± 15.2 years). Exploratory and confirmatory factor analysis was performed to examine the factor structure of the HAS-J. Cronbach’s *α* and McDonald’s *ω* were then used to test internal consistency. To examine the scale’s validity, we determined correlations between the HAS-J and other indexes and compared HAS-J scores between insomnia patients and community dwellers. We also compared HAS-J scores between two community-dweller groups (normal and poor sleepers) and two insomnia patient groups (with and without alleviation after treatment).

**Results:**

Following exploratory and confirmatory factor analysis, a 20-item measure emerged comprising three factors: “Introspectiveness and Reactivity,” “Neuroticism,” and “Insomnia.” Confirmatory factor analysis showed a generally good fit for the model of the three-factor structure suggested by the exploratory factor analysis loadings (*χ*^*2*^ (163) = 327.423, (*p* <  0.001), CFI = 0.914, GFI = 0.872, AGFI = 0.835, RMSEA = 0.067). In insomnia patients, internal consistency indicated sufficient reliability of the HAS-J. Correlation analysis showed weak to moderate positive correlations of the HAS-J score with other indexes, indicating concurrent validity of the HAS-J. All HAS-J subscale scores were significantly higher in insomnia patients than in community dwellers. Additionally, the total score in patients with alleviation of insomnia was comparable to that in poor sleepers and significantly higher than that in normal sleepers.

**Conclusions:**

This study demonstrated the reliability and validity of the HAS-J, indicating that it is useful as a clinical scale of hyperarousal. The high level of hyperarousal in insomnia patients who were assessed to be in remission by the Insomnia Severity Index suggests a risk of insomnia recurrence in these patients.

**Supplementary Information:**

The online version contains supplementary material available at 10.1186/s12888-022-04243-0.

## Introduction

Insomnia is characterized by nighttime symptoms of insomnia and consequent daytime dysfunction (or severe distress), both of which are required for diagnosis [1, 2]. Epidemiological studies have estimated the prevalence of insomnia to be around 20% in the general population of developed countries [3, 4]. Insomnia is associated with depression [5, 6], physical diseases such as cardiovascular disease [7, 8], and an increased risk of type 2 diabetes [9, 10], as well as deteriorated health-related quality of life [11–13].

Insomnia patients present not only with nighttime symptoms of insomnia, but also several daytime psychological and somatic conditions such as tension, anxiety, fatigue, and irritation. The physiological phenomena associated with elevated alertness that underlie both nighttime and daytime symptoms are sympathetic nervous tone, high-frequency activity on electroencephalography (EEG), and elevated metabolic rate, temperature, heart rate, and nighttime levels of blood cortisol and adrenocorticotrophic hormone [14, 15]. In the hyperarousal model of insomnia proposed by Riemann et al., the experience of chronic insomnia is a possible factor influencing the development of depression, addiction, and anxiety disorders [16]. On the other hand, the personality style of insomnia patients has been characterized by the internalization of psychological disturbances [17]. This suggests that the internalization causes emotional arousal and consequently physiological activation, which is a psychophysiological mechanism underlying insomnia. Previous findings have shown that hyperarousal is also associated with chronic stress [18], insomnia [19–23], cognitive anxiety [24], obstructive sleep apnea syndrome [25], metabolic syndrome, type 2 diabetes, and cardiovascular diseases [26–28]. Moreover, insomnia patients have shown changes in brain activity indicative of hyperarousal [16, 29]. A study using positron emission tomography revealed that, from waking to sleeping, insomnia patients had smaller decreases in metabolic activity in brain regions associated with arousal (e.g., the ascending reticular activating system, thalamus, hypothalamus, amygdala, hippocampus, and prefrontal cortex) compared with healthy individuals [30], indicating the neurological basis of hyperarousal in insomnia patients. Taken together and given the genetic, environmental, behavioral, and physiological factors involved in the causation and pathophysiology of insomnia [31], it is of clinical significance to determine the level of hyperarousal as an indicator of the vulnerability to insomnia and depression.

The self-reported Hyperarousal Scale (HAS) can be used to assess the level of cognitive and emotional hyperarousal. It was developed in relation to the Eysenck Personality Inventory [32], the Center for the Study of Epidemiology Depression Scale [33], the Coping Inventory for Stressful Situations Scale, and the Somatization Sensation Inventory [34]. The HAS provides scores for self-reported traits of cognitive and emotional hyperarousal in response to stimuli during wakefulness (i.e., tendency for introspection and responsiveness to stimuli) [20, 21, 23, 35]. These traits can be the causes of insomnia or vulnerability to insomnia [36]. The HAS has been evaluated in insomnia patients in relation to cortical auditory evoked potential, EEG spectral band activities [23], and actigraphy [21]. Also, significant decreases in HAS score were reported after cognitive behavioral therapy for insomnia [37].

To our knowledge, there are three versions of the HAS: the original US version, the Swedish version, and the Italian version. The Swedish and Italian versions have been confirmed to be reliable and valid in non-clinical groups of adults aged 40 years or older [38] and adults aged 18–80 years [39], respectively. Although these studies demonstrated that the HAS was negatively correlated with health-related quality of life [38, 39] and positively correlated with depression [38] and anxiety [39], there have been no relevant studies involving clinical groups. This simple self-reported scale is likely to serve as an index of vulnerability in insomnia patients and of risk factors for the recurrence of insomnia and depression. However, a similar scale is not available for use in Japan. In addition, some of the 26 items of the HAS have been extracted as subscales (Introspectiveness score, 6 items; Reactivity score, 3 items), but previous studies have not provided findings concerning the factor structure of the HAS using all items [35, 40]. To further advance the research on hyperarousal, it is necessary to clarify the structure of the full-item scale. There is also a measure called the Ford Insomnia Response to Stress Test (FIRST) [36], which measures insomnia vulnerability due to stress. The FIRST is a self-reported scale that assesses hyperarousal as insomnia vulnerability due to stress and asks about sleeping difficulties related to nine stressful experiences such as “after a stressful experience in the evening” and “before an important meeting the next day.” In contrast, the HAS characteristically not only assesses environments that hinder sleep, but also consists of a wide range of items relating to personality and other traits.

Therefore, the first objective of this study was to develop a Japanese version of the HAS (HAS-J) to be used with the Japanese population and investigate its factor structure using exploratory factor analysis (EFA) and confirmatory factor analysis (CFA) and then its reliability and validity. To examine its reliability, we asked insomnia patients to complete the HAS-J and we calculated Cronbach’s *α* and McDonald’s ω as a measure of its internal consistency. To examine its validity, we determined correlations of the HAS-J score with severity of insomnia and vulnerability to insomnia to indicate its criterion-related validity. This study tested the hypothesis that the HAS-J has moderate positive correlations with the Insomnia Severity Index (ISI), Athens Insomnia Scale (AIS), and FIRST, by which concurrent validity would be confirmed, and we compared HAS-J scores between insomnia patients and community dwellers. The second objective of this study was to calculate a cutoff score for the HAS-J. We also assessed different levels of hyperarousal in insomnia patients and community dwellers with and without insomnia symptoms to investigate the clinical utility of the HAS-J.

## Methods

### Participants

Insomnia patients: We recruited patients aged 20 years or older who had a diagnosis of chronic insomnia in accordance with ICSD-3 [1], were taking hypnotics, and were attending an outpatient sleep or psychiatric clinic at one of six medical facilities in Japan. We excluded patients with a sleep disorder other than insomnia (e.g., sleep apnea syndrome or periodic limb movement disorder). In total, 224 patients who met the eligibility criteria participated in this study. Mean age of the patients was 51.7 ± 15.6 years (range 20–86 years). There were 98 men (43.8%) and 126 women (56.3%). Mean duration of hypnotic use was 9 ± 7.4 years.

Community dwellers: We recruited individuals aged 20 years or older and without experience of shiftwork through advertising media distributed in Tokyo and its surrounding areas. We excluded individuals receiving treatment for psychiatric disorders (mood disorders, anxiety disorders, or sleep disorders) or taking hypnotics. There were 303 participants in total, comprising 128 men (42.2%) and 175 women (57.8%). Mean age of these participants was 43.9 ± 15.2 years (range: 23–76 years). Their occupations were as follows: information and communications (10.6%, *n* = 32), manufacturing (10.2%, *n* = 31), medical and social welfare (7.6%, *n* = 23), services (security, hairdressing, travel, postal) (6.3%, *n* = 19), education and learning support (5.9%, *n* = 18), and other (e.g., homemaker, unemployed, and student) (44.0%, *n* = 133). The most common pre-existing diseases besides “Other” were hypertension (10.6%, *n* = 32), diabetes (3.6%, *n* = 11), and asthma (2.3%, *n* = 7) (Supplementary 1).

### Materials

HAS: The original self-administered 26-item HAS rates the level of hyperarousal on a 4-point scale (0 = not at all, 1 = a little, 2 = quite a lot, and 3 = extremely). The total summation score has a range of 0–78. In addition, 6 items give an Introspectiveness score with a range of 0–18; 3 items give a Reactivity score with a range of 0–9; and the total number of items checked as ‘Extremely’ give an Extreme responses score. The Introspectiveness score is the sum of the following items: “My mind is always going,” “I think a lot about feelings,” “I tend to anticipate problems,” “I take things personally,” “Some thoughts return too often,” and “I take a long time to make decisions.” The Reactivity score is the sum of the following hyperarousal items: “Bright lights, crowds, noises or traffic bother me,” “I get rattled when a lot happens at once,” and “A sudden loud noise would cause me a prolonged reaction.” A higher score indicates a higher level of hyperarousal. However, it should be noted that factor structure for the full-item scale has not been reported for the original, Swedish, or Italian version of the HAS.

Creation of the Japanese version of the HAS: The original version of the HAS was translated into Japanese by two bilingual English–Japanese speakers who are specialists in the field, and the translated documents were independently back-translated by a native English speaker. The Japanese version was then further refined by Quentin Regestein who developed the original HAS and an American resident who is fluent in Japanese (Supplementary 2).

Severity of insomnia: We used two self-administered measures to assess severity of insomnia: the ISI and the AIS. The 7-item ISI is a 5-point scale (total score 0–28), and a higher score indicates more severe insomnia. The total score is calculated as follows: 0–7, no clinically significant insomnia; 8–14, sub-threshold insomnia; 15–21, moderate insomnia; and 22–28, severe insomnia. The Japanese version of the ISI has been shown to have high internal consistency (*α* = 0.84), and its reliability and validity have been verified by Munezawa et al., [41]. Given that an ISI cutoff score of 10 has been used in the assessment of a community population in previous research [42], we used the same cutoff score in the present study.

The 8-item AIS was developed as a measure to assess the severity of insomnia during the past month, based on the ICD-10 criteria [43, 44]. The items are evaluated using a 4-point Likert scale (0 = no problem at all to 3 = very severe problem), with a total score in the range of 0–24. A higher score indicates more severe insomnia. The Japanese version of the AIS consisted of a two-factor structure: “nocturnal sleep problem” (items 1–5) and “daytime dysfunction” (items 6–8). Internal consistency coefficients ranged from 0.78 to 0.88. A cutoff score of 5.5 was used in both the original and Japanese versions [43, 44].

Insomnia vulnerability to stress: The FIRST is a self-administered questionnaire that assesses the risks of sleep problems in response to stress [36]. The 9-item questionnaire uses a 4-point scale (1 = not likely to 4 = very likely). Total score ranges from 9 to 36, and a higher score indicates higher vulnerability. Cronbach’s *α* coefficients of the Japanese version of FIRST were 0.89 in insomnia patients and 0.87 in healthy subjects [45]. Because this measure assesses vulnerability to sleep reactivity, the validity of the HAS-J was evaluated by investigating its correlation with the FIRST.

### Statistical analysis

Since validation of the reliability and validity of the HAS original version had been tested with insomnia patients [20], the factor structure was investigated with insomnia patients in the present study as well. First, EFA using the maximum likelihood estimation, promax rotation, and scree plots was performed to extract latent factors. The Kaiser-Meyer-Olkin (KMO) measure and Bartlett’s test for sphericity were conducted to determine whether the collected data were suitable for factor analysis. In CFA, maximum likelihood estimation was used to estimate the CFA model, and the following fit indices were calculated: *χ*^*2*^ statistics (*p* value); comparative fit index (CFI); goodness-of-fit index (GFI); adjusted goodness-of-fit index (AGFI); and root mean square error of approximation (RMSEA). Next, Cronbach’s *α* and McDonald’s *ω* were used to test internal consistency [46]. To examine the validity of the HAS-J, correlation analysis was used to examine positive correlations between HAS-J scores and ISI, AIS, and FIRST scores for insomnia patients and community dwellers, respectively, and the HAS-J scores of insomnia patients and community dwellers were compared using a t-test. In addition, receiver operating characteristic (ROC) analysis was performed to calculate the cutoff score. Furthermore, to examine the clinical utility of the HAS-J, insomnia patients undergoing treatment and community dwellers were both divided based on insomnia score and the manifestations of insomnia based on HAS score were examined using multivariate analysis of variance. Statistical analyses were performed using IBM SPSS Statistics 28 and Amos 26 (Japan IBM, Tokyo, Japan) software. Statistical significance was set at *p* <  0.05.

## Results

### EFA of the HAS-J

We asked 224 patients with psychiatric illnesses presenting with insomnia symptoms to respond to the 26-item Japanese version of the HAS, and we then performed EFA. In this study, the KMO measure was .880 and Bartlett’s sphericity was statistically significant (*χ*^*2*^ = 2452.00, *df* = 325, *p* <  0.001), indicating that samples met the criteria for factor analysis. In EFA, factor loadings of 0.35 or better were used for interpretation purposes. Six items (items 4, 6, 7, 8, 19, and 24) with factor loadings less than 0.35 were deleted (Table 1). Finally, 20 items with a three-factor structure for the HAS-J were determined through EFA, accounting for 46.56% of the total variance.

**Table 1:** Results for exploratory factor analysis of the HAS-J

			I	II	III	*M*	*SD*
**I. Introspectiveness and Reactivity**							
20.	When things go wrong, I tend to get depressed.		**0.94**	−0.07	−0.12	1.73	0.94
12.	I get rattled when a lot happens at once. (R)		**0.87**	−0.18	− 0.11	1.52	0.98
22.	Some thoughts return too often. (I)		**0.84**	0.01	0.03	1.74	0.96
26.	I keep thinking about the same things long after they happened.		**0.81**	0.11	0.02	1.63	0.99
23.	I take a long time to make decisions. (I)		**0.74**	−0.08	−0.06	1.38	0.97
9.	I tend to anticipate problems. (I)		**0.65**	0.09	0.06	1.40	0.97
17.	A sudden, loud noise would cause me a prolonged reaction. (R)		**0.57**	0.05	0.02	1.10	0.97
5.	I think a lot about feelings. (I)		**0.57**	0.22	0.09	1.89	0.93
11.	I take things personally. (I)		**0.54**	0.21	0.10	1.42	0.95
25.	I get tearful easily.		**0.44**	−0.08	0.07	0.98	0.97
21.	My routine is predictable.		**0.42**	0.09	−0.14	1.56	0.93
10.	My bedroom is a mess.		**0.40**	−0.33	0.18	0.87	0.97
**II. Neuroticism**							
1.	I am well organized.		−0.12	**0.86**	−0.20	1.40	0.88
3.	I am a very careful worker.		−0.11	**0.68**	0.11	1.63	0.86
15.	I am a cautious person.		0.06	**0.55**	0.08	1.53	0.91
13.	I am good at details.		0.00	**0.47**	−0.01	1.01	1.00
18.	I am overly conscientious.		0.30	**0.46**	−0.08	1.28	0.89
**III. Insomnia**							
14.	I have trouble falling asleep.		−0.22	−0.05	**0.79**	1.56	1.07
16.	In bed at night, my thoughts keep going.		0.15	0.18	**0.67**	1.46	1.08
2.	I am slow to awaken mornings.		0.13	−0.11	**0.43**	1.23	1.02
		**Factor correlation**	**I**	**II**	**III**		
		**I**	–	0.45	0.54		
		**II**		–	0.29		
		**III**			–		
**Excluded items**							
4.	My mind is always going. (I)		–	–	–	1.68	0.92
6.	Bright lights, crowds, noises or traffic bother me. (R)		–	–	–	1.22	1.02
7.	Evenings are my best time.		–	–	–	0.93	1.01
8.	I cannot take naps, even if I try.		–	–	–	1.21	1.14
19.	Caffeine affects me strongly.		–	–	–	0.77	0.95
24.	Alcohol makes me sleepy.		–	–	–	0.90	1.00

Exploratory factor analysis was performed to extract latent factors, using maximum likelihood estimation and promax rotation

(I) = Introspectiveness subscale of the original version of the Hyperarousal Scale, (R) = Reactivity subscale of the original version of the Hyperarousal Scale

### CFA of the HAS-J

**Fig. 1:**
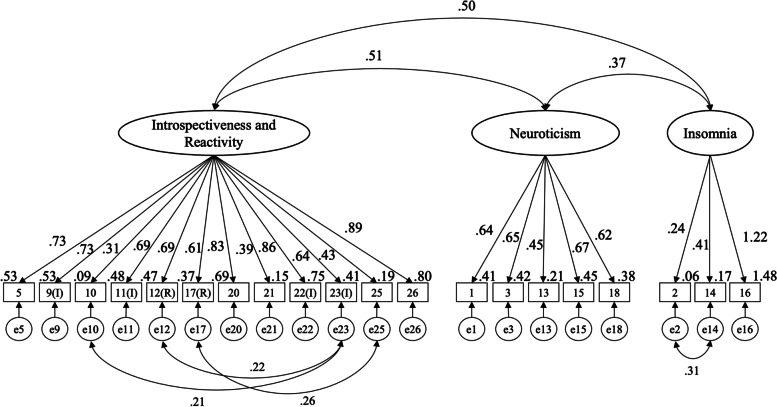
Confirmatory factor analysis of the HAS-J. Note. The numbers with unidirectional arrows are standard partial regression coefficients, the number in the upper right corner of the numbered square is the coefficient of determination (square of the multiple correlation coefficient), the number attached to the two-way arrow is the correlation coefficient and all of the paths are statistically significant with *p* values smaller than 0.01. Introspectiveness and Reactivity, Neuroticism, and Insomnia represent the subscales of the HAS-J. The numbers 1-26 represents the item numbers of the original version of Hyperarousal Scale, and e1-26 are all residual errors

The results of the Chi-square test for goodness-of-fit were first obtained and other indices were then assessed to determine the fit of the model. The fit indices of the model were as follows: *χ*^*2*^ (163) = 327.423 (*p* <  0.001); CMIN/DF = 2.01; CFI = 0.914; GFI = 0.872; AGFI = 0.835; AIC = 421.423; and RMSEA = 0.067 (90% CI = 0.057–0.078) (Fig. 1). All items were statistically significant at the *p* <  0.01 level.

The HAS-J ultimately comprised 12 items for Factor 1 “Introspectiveness and Reactivity,” 5 items for Factor 2 “Neuroticism,” and 3 items for Factor 3 “Insomnia.” The total score on the HAS-J has a range of 0–60. In addition, the 12 items for Factor 1 give an introspectiveness score with a range of 0–36; the 5 items for Factor 2 give a neuroticism score with a range of 0–15; and the 3 items for Factor 3 give an insomnia score with a range of 0–9. In line with previous studies, the total number of items checked as ‘Extremely’ was also used in determining reliability and validity in this study.

### Reliability

Internal consistency of the HAS-J was examined in the insomnia patients by calculating Cronbach’s *α* and McDonald’s *ω*. In the present study, Cronbach’s α (McDonald’s *ω*) coefficients were as follows: total score, 0.89 (0.89); Factor 1 “Introspectiveness and Reactivity,” 0.90 (0.91); Factor 2 “Neuroticism,” 0.74 (0.74); and Factor 3 “Insomnia,” 0.67 (0.67). Internal consistency was also examined in the community dwellers with the corresponding results: total score, 0.83(0.82); Factor 1 “Introspectiveness and Reactivity,” 0.83(0.84); Factor 2 “Neuroticism,” 0.64(0.65); and Factor 3 “Insomnia,” 0.63(0.69).

### Validity

To determine criterion-related validity of the HAS-J, we examined positive correlations between the HAS-J score and severity of insomnia on the AIS and ISI, as well as insomnia vulnerability to stress on the FIRST (Table 2). The AIS, ISI, and FIRST were not normally distributed and therefore Spearman’s correlation analysis was used. In the insomnia patient group, total score was moderately correlated with the FIRST and weakly correlated with the insomnia scales. Also, Factor 3 was moderately correlated with the ISI, AIS, and FIRST.

**Table 2:** Correlations coefficients of HAS-J scores with other indexes

	Insomnia patients (*n* = 224)			Community dwellers (*n* = 303)		
	ISI	AIS	FIRST	ISI	AIS	FIRST
Total score	0.32^**^	0.35^**^	0.67^**^	0.43^**^	0.45^**^	0.45^**^
F1: Introspectiveness and Reactivity	0.22^**^	0.27^**^	0.67^**^	0.40^**^	0.40^**^	0.42^**^
F2: Neuroticism	0.08	0.07	0.26^**^	0.00	0.07	0.16^**^
F3: Insomnia	0.59^**^	0.53^**^	0.48^**^	0.61^**^	0.59^**^	0.38^**^
Extreme responses	0.30^**^	0.32^**^	0.57^**^	0.17^**^	0.26^**^	0.19^**^

Total score = total score on the HAS-J (20 items), F1 = Factor 1 (12 items), F2 = Factor 2 (5 items), F3 = Factor 3 (3 items), Extreme responses = number of responses checked as ‘Extremely’

Abbreviations: *ISI* Insomnia Severity Index, *AIS* Athens Insomnia Scale, *FIRST* Ford Insomnia Response to Stress Test

*p <  0.05, **p <  0.01

When HAS-J scores were compared between the insomnia patients and community dwellers (Table 3), the insomnia patients had significantly higher scores on all subscales of the HAS-J (*p* <  0.01). Their mean ISI and AIS scores were 10.9 ± 6.0 and 7.4 ± 4.8, respectively, all of which exceeded the respective cutoffs.

**Table 3:** Comparison of HAS-J scores between insomnia patients and community dwellers

	Insomnia patients (*n* = 224)		Community dwellers (*n* = 303)			
	*M*	*SD*	*M*	*SD*	*t*	*p*
Total score	28.3	11.0	20.8	7.4	8.83	< 0.01
F1: Introspectiveness and Reactivity	17.2	8.0	12.5	5.4	7.62	< 0.01
F2: Neuroticism	6.8	3.2	6.1	2.5	2.94	< 0.01
F3: Insomnia symptoms	4.3	2.5	2.2	1.7	10.75	< 0.01
Extreme responses	3.8	4.5	1.1	1.9	8.54	< 0.01
ISI	10.9	6.0	5.4	3.9	12.05	< 0.01
AIS	7.3	4.8	4.3	3.1	8.38	< 0.01
FIRST	23.7	6.2	20.8	6.0	5.44	< 0.01

Total score = total score on the HAS-J (20 items), F1 = Factor 1 (12 items), F2 = Factor 2 (5 items), F3 = Factor 3 (3 items), Extreme responses = number of responses checked as ‘Extremely’

Abbreviations: *M* mean, *SD* standard deviation, *ISI* Insomnia Severity Index, *AIS* Athens Insomnia Scale, *FIRST* Ford Insomnia Response to Stress Test

### HAS-J cutoff score

**Fig. 2:**
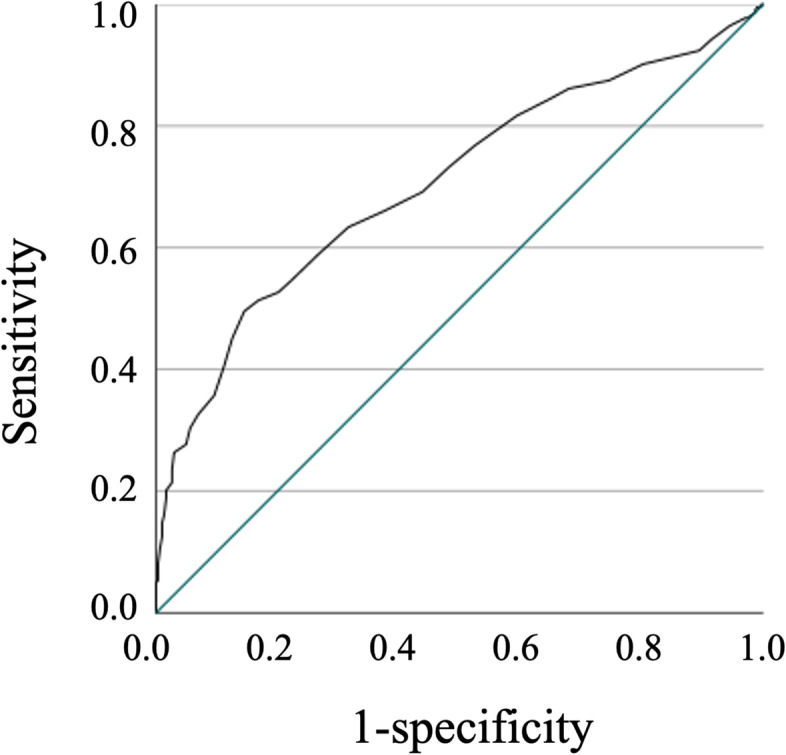
ROC curves for the total score on the HAS-J that were used to identify subjects with insomnia. Note. The AUC was 0.71 (0.66-0.75) and the cutoff score was 28.5. Sensitivity was 0.50 and specificity 0.85. The positive and negative predictive values were 71.6% and 69.6%, respectively

ROC analysis was used to calculate the HAS-J cutoff indicating a diagnosis of insomnia. Fig. 2 shows the ROC curves of the HAS-J total score, which were constructed for identifying insomnia patients. The area under the curve was 0.71 (95%CI: 0.66–0.75) and the cutoff score was 28.5. Sensitivity was 0.50 and specificity 0.85. The positive and negative predictive values were 71.6 and 69.6%, respectively.

### Relationship between severity of insomnia and HAS-J score

**Fig. 3:**
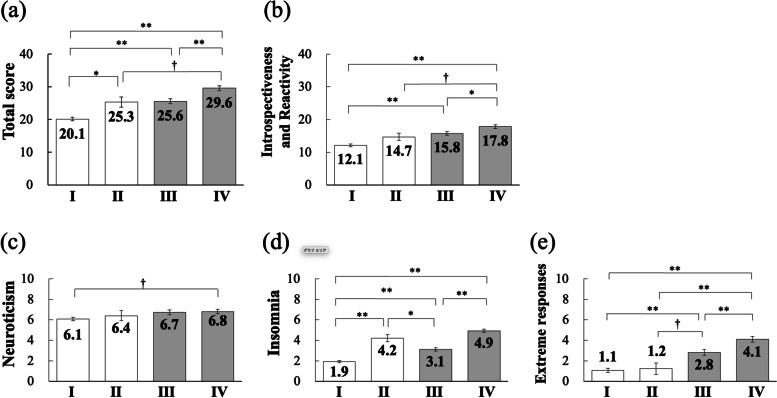
Differences in hyperarousal level according to the presence and absence of insomnia between community dwellers (I, normal sleepers; II, poor sleepers) and insomnia patients (III, with alleviation; IV, without alleviation). Note. Multivariate ANOVA with age covariates to analyze HAS-J scores (Bonferroni post-hoc tests). **a** Total score on the HAS-J, (**b**) Introspectiveness and Reactivity, (**c**) Neuroticism, (**d**) Insomnia, (**e**) Extreme responses. Error bars indicate standard error. †*p* < 0.10, *p < 0.05, **p < 0.01

We divided the community dwellers and insomnia patients into two groups each according to the ISI cutoff score of 10: normal sleepers (*n* = 261; mean ISI =4.2 ± 2.5) and poor sleepers (*n* = 42; mean ISI = 12.9 ± 2.3); and patients with alleviation of insomnia after treatment (*n* = 104; mean ISI = 5.7 ± 2.6) and those without alleviation of insomnia after treatment (*n* = 120; mean ISI = 15.5 ± 4.1), respectively. To examine differences in hyperarousal level according to the presence and absence of insomnia symptoms, multivariate analysis of variance was performed with HAS-J score as the dependent variable, the four groups as independent variables, and age as a covariate. Bonferroni’s multiple comparison test showed that, in patients with alleviation after treatment, the ISI score was below the cutoff, but the total score was comparable to that of poor sleepers among the community dwellers (Fig. 3).

## Discussion

This study is the first to examine the HAS in an Asiatic population, specifically Japanese insomnia patients and community dwellers. The first objective of this study was to develop a Japanese version of the HAS to be used with the Japanese population and to investigate its factor structure, reliability, and validity. The second objective of the study was to calculate a cutoff score for the HAS-J and examine its clinical utility. We also assessed different levels of hyperarousal in insomnia patients and community dwellers with and without insomnia symptoms to investigate the clinical utility of the HAS-J.

This study is also the first to determine the factor structure of the HAS. Following EFA, a 20-item measure emerged comprising three factors: “Introspectiveness and Reactivity,” “Neuroticism,” and “Insomnia.” CFA showed a generally good fit except for the *χ*^*2*^ statistic, which was sensitive to sample size for the model with the three-factor structure suggested by the EFA. The two subscale factors used in the original version of the HAS (6-item Introspectiveness scale and 3-item Reactivity scale) were not found in this study, and the new three-factor structure was adopted based on the EFA and CFA results. Except for the two items that had factor loadings of less than 0.35 in the EFA (one of the Reactivity items, “6. Bright lights, crowds, noises or traffic bother me” and one of the Introspectiveness items, “4. My mind is always going”), all 7 remaining items were found to be included in Factor 1. This means that the Introspectiveness and Reactivity items extracted from the original version are included in Factor 1 of the HAS-J, “Introspectiveness and Reactivity”, and may be described as the principal content of hyperarousal. In comparison with hyperarousal assessed using the FIRST, which reflects insomnia vulnerability due to stress, these 20 hyperarousal items shown in these three factors may be regarded as a concept resembling the symptom of hyperawareness in post-traumatic stress disorder.

Six of the 26 items were eliminated from the HAS-J (item 4: “My mind is always going”; item 6: “Bright lights, crowds, noises or traffic bother me”; item 7: “Evenings are my best time”; item 8: “I cannot take naps, even if I try”; item 19: “Caffeine affects me strongly”; and item 24: “Alcohol makes me sleepy”). For three of these items (items 7, 19, and 24), a floor effect was observed. The reasons for this are not clear, but it is possible that patients with insomnia who take hypnotic medications may have intentionally refrained from consuming alcohol and caffeine, which may have resulted in disproportionately low response scores. Consequently, at least in the results for Japanese insomnia patients, these items were not sensitive detectors of hyperarousal. However, it is not necessarily expected that similar results would be obtained in other populations. Further study is warranted.

Reliability of the HAS-J was evaluated using Cronbach’s *α* and McDonald’s *ω*. A reliability coefficient greater than 0.7–0.8 is generally considered to show sufficient internal validity [47–49]. In the present study, although the coefficients were slightly lower for Factor 3 “Insomnia” in insomnia patients, the reliability of the HAS-J is considered sufficient. The validity of the HAS-J was assessed using other indexes, namely, the ISI, AIS, and FIRST. The HAS-J was found to have a moderate positive correlation with stress vulnerability (FIRST) and a weak to moderate positive correlation with the insomnia scales (ISI, AIS). Factor 3 “Insomnia” is a subscale associated with insomnia and therefore naturally had a high correlation with the ISI and AIS compared with the other two HAS-J factors. This study also found that insomnia patients had significantly higher scores than community dwellers on all subscales of the HAS-J, indicating they had high hyperarousal levels, which is in good agreement with previous studies [20, 23, 35]. Thus, our hypothesis was supported, and the validity of the HAS-J was indicated.

It is interesting to note that the HAS-J score was still significantly higher in insomnia patients with alleviation of insomnia than in normal sleepers and was comparable to that in poor sleepers among community dwellers. These results indicate that insomnia patients in this study were hyperaroused even though they were in remission after treatment, and thus they are at risk of recurrent insomnia. This suggests that not only is assessment of hyperarousal status useful in determining the severity of insomnia based on the presence/absence of insomnia symptoms, but also that it has clinical value in assessing the risks of onset and recurrence. On the other hand, poor sleepers, who presumably had subclinical insomnia and were not seeking medical care or taking any medication, could be a group at risk of insomnia and depression upon exposure to stress.

In addition to the above analysis, the cutoff score of the HAS-J was calculated using ROC analysis to indicate the diagnosis of insomnia. The HAS-J cutoff score for distinguishing people with insomnia from healthy individuals was previously unknown. ROC curves for Japanese individuals in this study showed a cutoff score of 28.5. However, given the relatively low ISI scores in insomnia patients in this study, the cutoff could be higher if different patient cohorts are tested. The cutoff score will also vary in different groups of patients, for example, those with depression or other comorbidities, and it will also depend on the severity of insomnia.

There are several limitations to this study. First, the community dwellers were not randomly selected and thus do not necessarily represent the general population. Information on background factors, such as character traits, was limited. Second, the HAS is self-administered, meaning that responses are based on participants’ subjective views. Therefore, it is impossible to rule out the possibility that participants intentionally manipulated their responses or that they responded based on an insufficient understanding of the items and/or themselves. Third, this was cross-sectional study, which precludes discussion of causal relationships. As such, we cannot rule out common method variance (i.e., variance attributable to the measurement method rather than to the constructs represented by the measurement method) [50]. The results of this study must be interpreted with the underlying assumption that such a bias may exist. Fourth, CFA was performed using the same insomnia patient data used for EFA. Future studies should perform CFA with a different insomnia patient cohort in order to confirm the structure of the HAS-J. Even with these limitations, the results of this study suggest that the HAS-J is a useful screening instrument to detect Japanese individuals who are vulnerable to mood disorders and insomnia. A prospective observational study and an interventional study are warranted to evaluate clinical use of the HAS-J in predicting the risks of onset and recurrence of depression and insomnia.

## Conclusion

This study revealed the factor structure and confirmed the reliability and validity of the HAS-J, indicating that it is a useful clinical scale of hyperarousal. The high level of hyperarousal seen in insomnia patients who were assessed to be in remission by the ISI suggests a risk of insomnia recurrence in these patients.

## Supplementary Information

**Additional file 1.**

**Additional file 2.**

## Data Availability

The datasets generated and/or analyzed during the current study are not publicly available because the data belong to the Ministry of Health, Labour and Welfare of Japan, but are available from the corresponding author on reasonable request.
