# Supplementary material for: Development and validation of the Japanese version of the Hyperarousal Scale
Source: BMC Psychiatry. 2022 Sep 19;22:617. doi: 10.1186/s12888-022-04243-0 (PMC9484233; doi:10.1186/s12888-022-04243-0)
Supplement: Supplementary file 1 — Additional file 1. [file 12888_2022_4243_MOESM1_ESM.docx]

Supplementary 1 Demographic data of community dwellers (*n* = 303).

|  |  |  | | |  |
| --- | --- | --- | --- | --- | --- |
|  |  |  | *M* | *SD* |  |
|  | Age | | 43.9 | 15.2 |  |
|  | Women (%) | | 175 (57.8) |  |  |
|  | Instrument | |  |  |  |
|  |  | ISI | 5.4 | 3.9 |  |
|  |  | AIS | 4.3 | 3.1 |  |
|  |  | FIRST | 20.8 | 6.0 |  |
|  | Occupation | | *n* | % ^a^ |  |
|  |  | Agriculture, forestry and fisheries | 4 | 1.3 |  |
|  |  | Construction | 3 | 1.0 |  |
|  |  | Manufacturing | 31 | 10.2 |  |
|  |  | Information and communications | 32 | 10.6 |  |
|  |  | Transport | 4 | 1.3 |  |
|  |  | Wholesale and retail | 11 | 3.6 |  |
|  |  | Finance and insurance | 10 | 3.3 |  |
|  |  | Real estate | 2 | .7 |  |
|  |  | Hospitality | 3 | 1.0 |  |
|  |  | Medical and social welfare | 23 | 7.6 |  |
|  |  | Education, learning support | 18 | 5.9 |  |
|  |  | Services (security, hairdressing, travel, postal) | 19 | 6.3 |  |
|  |  | Government service | 10 | 3.3 |  |
|  |  | Other (e.g., homemaker, unemployed, and student) | 133 | 44.0 |  |
|  | Preexisting condition ^b^ | | *n* | % ^a^ |  |
|  |  | Gastrointestinal disease | 4 | 1.3 |  |
|  |  | Hypertension | 32 | 10.6 |  |
|  |  | Heart disease | 5 | 1.7 |  |
|  |  | Diabetes | 11 | 3.6 |  |
|  |  | Liver disease | 2 | .7 |  |
|  |  | Asthma | 7 | 2.3 |  |
|  |  | Cancer | 4 | 1.3 |  |
|  |  | Other | 43 | 14.2 |  |
|  |  |  |  |  |  |

*Note.*　a: percentage (%) of 303 subjects, b: Multiple answers allowed.

Abbreviations: M = mean, SD = standard deviation, ISI = Insomnia Severity Index, AIS = Athens Insomnia Scale, FIRST = Ford Insomnia Response to Stress Test.
