# Supplementary material for: Development and validation of the Japanese version of the Hyperarousal Scale
Source: BMC Psychiatry. 2022 Sep 19;22:617. doi: 10.1186/s12888-022-04243-0 (PMC9484233; doi:10.1186/s12888-022-04243-0)
Supplement: Supplementary file 2 — Additional file 2. [file 12888_2022_4243_MOESM2_ESM.docx]

Supplemental 2 Original and Japanese versions of the Hyperarousal Scale.

| Item | Original Version | Japanese Version |
| --- | --- | --- |
|  | **Check each item as it best applies to you.** | **あなたに最もよく当てはまる回答を選択してください。** |
| 1. | I am well organized. | 私は、几帳面だ |
| 2. | I am slow to awaken mornings. | 私は、朝は目覚めが悪い |
| 3. | I am a very careful worker. | 私は、注意深く仕事をする |
| 4. | My mind is always going. (I) | 私は、いつも考え事をしている |
| 5. | I think a lot about feelings. (I) | 私は、自分や他人の感情を気にしがちだ |
| 6. | Bright lights, crowds, noises or traffic bother me. (R) | 私は、明るい光、人混み、騒音、車の行き来が煩わしい |
| 7. | Evenings are my best time. | 私は、夕方から夜にかけてが最も好きな時間帯だ |
| 8. | I cannot take naps, even if I try. | 私は、昼寝はしようと思ってもできない |
| 9. | I tend to anticipate problems. (I) | 私は、問題が起こると思いがちだ |
| 10. | My bedroom is a mess. | 私の寝室は散らかっている |
| 11. | I take things personally. (I) | 私は、何事も自分自身に関連づけて考える |
| 12. | I get rattled when a lot happens at once. (R) | 私は、たくさんの事が同時に起こると混乱してしまう |
| 13. | I am good at details. | 私は、細かい作業が得意だ |
| 14. | I have trouble falling asleep. | 私は、寝つきが悪い |
| 15. | I am a cautious person. | 私は、用心深い人間だ |
| 16. | In bed at night, my thoughts keep going. | 私は、夜、寝床に入っても考え続けてしまう |
| 17. | A sudden, loud noise would cause me a prolonged reaction. (R) | 私は、突然、大きな音を聞いたら、ドキドキがなかなか収まらないだろう |
| 18. | I am overly conscientious. | 私は、過度に実直だ |
| 19. | Caffeine affects me strongly. | 私は、カフェインが非常に良く効く |
| 20. | When things go wrong I tend to get depressed. | 私は、物事がうまくいかないと落ち込みやすい |
| 21. | My routine is predictable. | 私の日常の活動はいつもほぼ同じである |
| 22. | Some thoughts return too often. (I) | 私は、何度も何度も同じ事を考え込んでしまう |
| 23. | I take a long time to make decisions. (I) | 私は、決断するのに時間がかかる |
| 24. | Alcohol makes me sleepy. | 私は、飲酒すると眠くなる |
| 25. | I get tearful easily. | 私は、些細なことで涙が出る |
| 26. | I keep thinking about the same things long after they happened. | 私は、いつまでも同じ事が頭から離れない |

*Note.* (I)＝Introspectiveness subscale of the original version of the Hyperarousal Scale, (R)＝Reactivity subscale of the original version of the Hyperarousal Scale.
